# Comparative Effectiveness of Cytisinicline and Varenicline for Smoking Cessation: A Matching-Adjusted Indirect Comparison (MAIC)

**DOI:** 10.36469/001c.160017

**Published:** 2026-05-20

**Authors:** Mark Rubinstein, David Rowe, Renee Perdok, Cindy Jacobs

**Affiliations:** 1 Achieve Life Sciences, Seattle, Washington; 2 Medical Decision Modeling Inc., Indianapolis, Indiana

**Keywords:** comparative effectiveness, cytisinicline, indirect treatment comparison (MAIC), nausea, smoking cessation, varenicline

## Abstract

**Background:**

Pharmacologic options for supporting smoking cessation remain limited. Cytisinicline is a plant-based alkaloid that selectively binds to α4β2 nicotinic acetylcholine receptors, reducing nicotine craving and withdrawal symptoms, and has proven effective for smoking cessation in the phase III ORCA-2 and ORCA-3 trials, with a favorable safety profile. Using data from randomized clinical trials, we performed an indirect treatment comparison of varenicline, a guideline-recommended partial agonist of the nicotinic acetylcholine receptor used as first-line pharmacotherapy for tobacco cessation, vs cytisinicline for smoking cessation.

**Objectives:**

This analysis was designed to compare the efficacy and safety of cytisinicline vs varenicline using pooled data from the cytisinicline ORCA-2 and ORCA-3 trials, and the EAGLES trial of varenicline.

**Methods:**

ORCA-2 (conducted from October 2020 to December 2021), ORCA-3 (May 2023–March 2024), and EAGLES (November 2011–January 2015) enrolled adults who smoked at least 10 cigarettes daily and were motivated to quit. A matching-adjusted indirect treatment comparison was employed to adjust for patient population differences between the ORCA (cytisinicline) and EAGLES (varenicline) studies. Propensity score weighting was used to align patient characteristics, then efficacy outcomes, continuous smoking abstinence rate at Weeks 9-12 and Weeks 9-24, and safety outcomes, including treatment discontinuation due to adverse events, were assessed. Adjusted and unadjusted analyses (a standard Bucher and the Mantel-Haenszel approach) were performed.

**Results:**

There was no statistically significant difference in continuous abstinence between the cytisinicline and varenicline cohorts at the 9- to 12-week assessment (adjusted OR, 1.33; 95% CI, 0.86-2.05). Patients prescribed cytisinicline had higher odds of abstinence vs varenicline at the 9- to 24-week assessment (adjusted OR, 1.95; 95% CI, 1.13-3.34). Cytisinicline was associated with 82% lower odds of nausea vs varenicline (adjusted OR, 0.18; 95% CI, 0.10-0.31).

**Conclusion:**

Cytisinicline may be an effective and more tolerable alternative, especially for patients at risk of relapse or unable to tolerate varenicline.

## INTRODUCTION

Cigarette smoking remains the leading cause of preventable disease, disability, and death in the United States, responsible for over 480 000 deaths annually.^1^ For every day a smoker continues, their life expectancy is shortened by an estimated 4 to 6 hours.^2,3^

Attempting to quit smoking without pharmacologic or nicotine replacement support has a high failure rate of around 95%, mainly due to the associated nicotine withdrawal symptoms and cravings.^4^ Nicotine replacement therapy and the pharmacologic agents varenicline and bupropion have been shown to reduce withdrawal symptoms, and their use is associated with improved smoking cessation success rates compared with unsupported quit attempts.^5–11^ Beyond reducing withdrawal, smoking cessation pharmacotherapies can reduce cravings and block the perceived reward from smoking.^12,13^

### Treatment Options and Unmet Needs for Smoking Cessation

Before successfully quitting cigarette smoking, people often have several failed attempts, with almost 80% of those who try to quit relapsing within 6 months.^14,15^ Treatment failure can undermine the motivation to try quitting again.^16–18^ For those experiencing failure to quit smoking, access to effective pharmacologic support can be critical for future success.^19^ Current treatments vary in their mode of action and adverse event profiles; new treatments are needed to offer effective and tolerable support to people in their efforts to quit smoking.^19^

Even with pharmacologic support, successful quit rates vary from approximately 20%^6^ with bupropion, transdermal nicotine, or nicotine gum to 14.4% to 44.0% with varenicline.^5–11,20–23^ However, varenicline is associated with a number of troublesome side effects, commonly including nausea and vomiting, weight gain, dizziness, headache, and sleep disturbances (in ≥1% of clinical trial participants), leading to treatment discontinuation (in 2%-53%) and relapse (in 25%).^24,25^

### Cytisinicline for Smoking Cessation

Cytisinicline is a highly selective, partial agonist of α4β2 nicotinic acetylcholine receptors (naChR) and has been shown to reduce cravings and withdrawal symptoms among individuals attempting to quit smoking.^26,27^ Historically, cytisinicline has been used for smoking cessation in Central and Eastern Europe, with 6 times daily dosing and down-titration over a 25-day treatment period. More recently, analyses of cytisinicline pharmacokinetics have enabled the development of a novel formulation and dosing regimen of 3 mg, 3 times a day (tid) for 6 or 12 weeks, to optimize efficacy while minimizing side effects and daily dosing burden.^26^ Phase III evaluations of this regimen in the Ongoing Research of Cytisinicline for Addiction (ORCA)-2 and ORCA-3 clinical trials have demonstrated the efficacy of cytisinicline as a smoking cessation treatment.^26,27^ ORCA-2 was a phase III, randomized, double-blind clinical trial that compared cytisinicline 3 mg tid, over 6 or 12 weeks, with placebo.^26^ ORCA-3 was a replica phase III study conducted to confirm the results of ORCA-2.^27^ Both the 6- and 12-week cytisinicline regimens were more effective than placebo in terms of higher continuous smoking abstinence rates, extended through to Week 24 in ORCA-3.^27^

While head-to-head clinical studies with varenicline have been conducted with the historical cytisine formulation using a 1.5 mg tablet administered in a 25-day downward titration regimen,^28–30^ no studies, to date, have directly compared varenicline with the novel cytisinicline formulation evaluated in the ORCA program (3 mg tid for 12 weeks), which is currently under regulatory review by the US Food and Drug Administration. In the absence of large head-to-head randomized controlled trials (RCTs), indirect treatment comparisons (ITCs) can offer insights into the relative safety and efficacy of treatments. Specifically, matching-adjusted indirect comparisons (MAICs) adjust for differences in patient populations across trials to provide meaningful comparisons relevant for clinical decision-making.^31,32^

This analysis was undertaken to compare the safety and efficacy of cytisinicline 3 mg tid with that of varenicline using pooled data from 2 large phase III trials of cytisinicline, ORCA-2^26^ and ORCA-3,^27^ and the EAGLES phase IV trial of varenicline.^33^ Varenicline was chosen as the comparator because it is also a partial agonist at the nicotinic acetylcholine receptor,^5^ is recommended as a first-line treatment across major international and national guidelines on smoking cessation,^11,34^ and has been studied in a large, recent, well-characterized phase IV trial (EAGLES).^33^

## METHODS

### Trials Overview and Participants

Efficacy and safety data were pooled from the 2 phase III trials of cytisinicline 3 mg tid, the ORCA-2 and ORCA-3 studies, and the nonpsychiatric cohort from the most recent and largest phase IV trial of varenicline, the EAGLES study.^26,27,33^ The full inclusion and exclusion criteria for ORCA-2 (conducted October 2020–December 2021), ORCA-3 (conducted May 2023–March 2024), and EAGLES (conducted November 2011–January 2015) have been described previously.^26,27,33,35^ In ORCA-2 and ORCA-3, adults from the US aged 18 years and older were eligible if they currently smoked at least 10 cigarettes per day, had expired air carbon monoxide (CO) levels at least 10 ppm, and were ready to set a date to quit smoking. Individuals were excluded if they used any noncigarette tobacco product, electronic cigarettes, medication, or marijuana in the 28 days (ORCA-2) or 14 days (ORCA-3) prior to randomization, or planned to use them during the trial.^26,27^ In EAGLES, adults 18 to 75 years were eligible if they smoked an average of at least 10 cigarettes per day during the last 12 months and during the month before the screening visit, had an exhaled CO greater than 10 ppm at screening, and were motivated to quit smoking.^33^ The EAGLES study included patients with/without psychiatric disorders, to assess neuropsychiatric safety risks of varenicline use. Because participants with a current psychosis or a recent psychotic episode were excluded in the ORCA studies, the nonpsychiatric cohort from the EAGLES study was used in the analysis reported here. Smokers with unstable psychiatric disorders or with current, active substance abuse were excluded from the secondary analysis of the EAGLES study due to small sample size and modeling interference.^35^ Several adjusted comparisons of the relative efficacy and safety of cytisinicline monotherapy (3 mg tid for 12 weeks) to varenicline monotherapy (1 mg twice daily for 12 weeks) were performed. The analyses used de-identified data under data use agreements.

### Efficacy and Safety Outcomes

The primary efficacy outcome was the biochemically confirmed continuous smoking abstinence rate from Weeks 9-12 and Weeks 9-24. Abstinence was defined as self-reported smoking cessation confirmed by expired CO levels. Safety outcomes included the treatment discontinuation rate due to treatment-emergent adverse events (TEAEs). The differences in occurrence of the 3 most reported TEAEs for both treatments (nausea, insomnia, and abnormal dreams) were also evaluated separately. In ORCA-2 and ORCA-3, safety was assessed at weekly visits during treatment (Weeks 1-12), and through an additional 12-week follow-up, by measurement of vital signs and participant self-report of adverse events and concomitant medications. Hematology and chemistry laboratory tests were conducted at Weeks 1, 6, and 12. Electrocardiograms were conducted at Weeks 6 and 12. Adverse events of abnormalities considered clinically significant were followed until resolution or end of study.^26,27^ In EAGLES, the primary safety outcome was a composite neuropsychiatric assessment; emergence of adverse events was assessed with open-ended questions, direct observation, and a semi-structured Neuropsychiatric Adverse Events Interview, completed at all study visits (up to 15 face-to-face visits and 11 telephone visits during the 24-week trial) by trained interviewers.^33^

### Assessment of Smoking Status

In the ORCA trials, smoking status was assessed weekly at Weeks 2-12 and defined as self-report of smoking abstinence since last visit.^26,27^ Self-reported abstinence at Weeks 16, 20, and 24 used the Russell Standard criteria of not smoking >5 cigarettes between Weeks 16 and 24. A breath CO <10 parts per million was required to confirm abstinence at each visit.

In the EAGLES trial, smoking cessation was defined as continuous abstinence for Weeks 9-12, self-reported and confirmed with an exhaled CO <10 parts per million. Smoking abstinence for Weeks 9-24 was similarly defined.^33^ Smoking abstinence was assessed weekly using a structured questionnaire at all clinic visits and telephone contacts (up to 15 face-to-face visits and 11 telephone calls took place during the 24-week trial).^33^

### Statistical Analyses

All efficacy results are reported as odds ratios (ORs). The primary analysis employed propensity score weighting to adjust selected covariates (race and cigarettes smoked per day in the past 30 days) in the pooled ORCA studies to match the EAGLES nonpsychiatric cohort. Unadjusted analyses used inverse-variance weighting for outcomes in both the pooled ORCA and the EAGLES nonpsychiatric cohort (ie, a standard Bucher analysis).^36^ Unadjusted ITCs of all outcomes were also conducted using the Mantel-Haenszel approach for dichotomous outcomes. This approach was selected as the preferred methodology for low-incidence events to reduce bias.^37^

The efficacy and safety outcomes were adjusted by reweighting the pooled ORCA data to align with the summary statistics of the nonpsychiatric EAGLES cohort. This adjustment was undertaken to account for differences in patient populations across trials thereby enabling meaningful comparisons and was based on prespecified treatment effect modifiers (see below) identified through a review of the literature regarding potential modifiers in the ORCA and EAGLES trials.^37^

Identifying treatment effect modifiers can prove to be challenging, particularly in analyses of novel therapies. In the primary analysis presented here, only variables that have indicated a potential to be treatment effect modifiers in previous analyses of varenicline or cytisinicline were considered.^26,27,35^ In secondary adjusted analyses, additional conceptually distinct variables reported by both the EAGLES nonpsychiatric cohort and the ORCA studies that demonstrated a strong prognostic effect or less convincing evidence of effect modifier status^26,27,35^ were also considered. Treatment effect modifiers were prespecified through a review of the published literature and clinical study reports from ORCA-2 and ORCA-3; these included age, race, and the Hospital Anxiety and Depression Scale (HADS; **Table 1**). The identification of treatment effect modifiers was informed by previous studies, including stepwise logistic regression analyses of the EAGLES study and neuropsychiatric adverse event data.^35^ Age, race, and the HADS were selected as treatment effect modifiers based on their established roles as prognostic factors and potential treatment effect modifiers in smoking cessation. Among older individuals (aged ≥60 years), those highly dependent on nicotine have poorer cessation outcomes than younger individuals (aged <60 years). However, among smokers unmotivated to quit, older individuals had better outcomes.^38^ Differences in quit rates across racial/ethnic groups may reflect economic and social determinants of health, including access to healthcare.^39^ Anxiety symptoms may be associated with increased nicotine dependence and increased risk of relapse.^40^

To address potential systematic bias due to study-level differences in treatment effect modifiers, propensity score weighting was used on individual patient data from the pooled ORCA studies. This approach matched the distribution of relevant treatment effect modifiers to the most comparable cohort in the EAGLES study, referred to as a MAIC. MAIC allows for the comparison of efficacy and safety across studies without assuming a fixed outcome scale, which reduces bias when analyzing nonlinear outcomes with aggregate data.

Three sets of potential treatment effect modifiers were considered. Each successive set includes additional potential treatment effect modifiers with less convincing evidence of effect modifier status in previous analyses (**Table 1**):

**Set 1: Race, cigarettes per day in the last 30 days:** This set included only the variables with the strongest evidence of effect modifier status for both varenicline and cytisinicline.

**Set 2: Set 1 + HADS anxiety score^41^:** This set included the potential effect modifiers of Set 1 in addition to the HADS anxiety score. The HADS anxiety score demonstrated the most potential as a treatment effect modifier in an analysis of neuropsychiatric adverse events in the EAGLES nonpsychiatric cohort, although there was very little evidence suggesting that potential effect modification existed in the current analysis.

**Set 3: Set 2 + age + HADS depression score^41^:** This set included all the potential effect modifiers of Set 2, in addition to age and the HADS depression score. Age and HADS depression score demonstrated significant prognostic effects in an analysis of potential treatment effect modifiers of the continuous smoking abstinence rate at Weeks 9-24 in the entire EAGLES cohort. Furthermore, these were conceptually distinct measures when compared with already included potential effect modifiers.

**Table 1.** Prespecified Treatment Effect Modifiers^26,27,35^

**Treatment Effect Modifier**	**Set**		
Race	} Set 1	} Set 2	$\}$ Set 3
Cigarettes smoked per day in the last month			
HADS anxiety score			
Age			
HADS depression score			

Abbreviation: HADS, Hospital Anxiety and Depression Scale.

To select propensity score weights for a given set, a logistic regression model using all selected treatment effect modifiers in that set was used. The standard error of the reweighted log OR (LOR) was estimated using a robust sandwich estimator. In the case of linear outcome models, MAICs are “doubly robust,” meaning the estimator is consistent if either the propensity score model or the outcome model is correctly specified. However, because this doubly robust property of MAICs does not generally hold for nonlinear outcome models, no outcome regression was performed on the reweighted data.^42^

This analysis used the traditional frequentist inverse-variance approach to compare efficacy and safety outcomes without adjustment for population-level differences. The treatment effect for pooled ORCA data was the pooled LOR of both ORCA trials using inverse-variance weighting. The comparisons were implemented in R using the package ‘netmeta.’^43^ The ‘netmeta’ package uses the graph-theoretical approach for network meta-analyses. This has been shown to be equivalent to the frequentist approach of least squares regression (ie, inverse-variance weighting).^44^ The LOR reported in the primary publication for EAGLES in the nonpsychiatric cohort was used to compare against the pooled cytisinicline LOR.

An additional analysis was conducted in which relative safety and efficacy comparisons were made using the Mantel-Haenszel approach to network meta-analyses. The Mantel-Haenszel approach has been shown to be less biased in cases where the rate of events is low.^37^ This may be particularly relevant for safety outcomes, because some safety outcomes are uncommon. For a given outcome, the observed number of events for each ORCA study, along with the observed number of events in the EAGLES study, were used as the input data. In cases where only the rate of events was reported, the numeric outcome count was approximated by multiplying the reported rate by the size of the relevant treatment arm. The implementation used the R package ‘netmeta’ and specifically used the function ‘netmetabin.’^43^

## RESULTS

### Efficacy Analyses

**Continuous smoking abstinence rate Weeks 9-12 (unadjusted):** For the continuous smoking abstinence rate Weeks 9-12 outcome, ORs (95% CI) for cytisinicline vs varenicline ranged from 1.30 (0.83-2.02) for inverse variance to 1.32 (0.87-2.02) for Mantel-Haenszel, with no significant differences (**Table 2**).

**Table 2.** Unadjusted Efficacy Results for Cytisinicline vs Varenicline

**Continuous Smoking Abstinence Rate, Weeks**	**Cytisinicline vs Varenicline^a^, OR (95% CI)**	
**Inverse Variance**	**Mantel-Haenszel**	
9-12	1.30 (0.83-2.02)	1.32 (0.87-2.02)
9-24	1.85 (1.06-3.24)*	1.92 (1.14-3.24)*

Abbreviations: CI, confidence interval; OR, odds ratio.

*P < .05.

^a^Nonpsychiatric cohort.

**Continuous smoking abstinence rate Weeks 9-12 (adjusted):** The adjusted ORs (aORs) for cytisinicline vs varenicline were consistent across all three sets of treatment effect modifiers (Set 1, Set 2, Set 3), with aORs ranging from 1.32-1.33, indicating no significant difference between the 2 treatments (**Table 3**).

**Table 3.** Adjusted Efficacy Results for Cytisinicline vs Varenicline

**Continuous Smoking Abstinence Rate, Weeks**	**Cytisinicline vs Varenicline^a^, aOR (95% CI)**		
**Set 1 (Primary)**	**Set 2**	**Set 3**	
9-12	1.33 (0.86-2.05)	1.32 (0.84-2.09)	1.32 (0.80-2.18)
9-24	1.95 (1.13-3.34)*	2.13 (1.23-3.69)*	1.90 (1.05-3.44)*

Abbreviations: aOR, adjusted odds ratio; CI, confidence interval; HADS, Hospital Anxiety and Depression Score.

Note: Selected treatment effect modifiers Set 1 (race, cigarettes per day in the last 30 days), Set 2 (Set 1 + HADS anxiety score), and Set 3 (Set 2 + age + HADS depression score).

*P < .05.

^a^Nonpsychiatric cohort.

**Continuous smoking abstinence rate Weeks 9-24 (unadjusted):** Both unadjusted models showed statistically significant results (inverse variance: OR, 1.85; 95% CI, 1.06-3.24; Mantel-Haenszel: OR, 1.92; 95% CI, 1.14-3.24), indicating superior efficacy of cytisinicline over varenicline for longer-term smoking abstinence.

**Continuous smoking abstinence rate Weeks 9-24 (adjusted):** Cytisinicline demonstrated a superior efficacy profile compared with varenicline, with aORs ranging from 1.90 to 2.13 across the three sets of treatment effect modifiers. Statistically significant results were observed for Set 1 (aOR, 1.95; 95% CI, 1.13-3.34), Set 2 (aOR, 2.13; 95% CI, 1.23-3.69), and Set 3 (aOR, 1.90; 95% CI, 1.05-3.44), suggesting that cytisinicline was superior to varenicline in promoting longer-term smoking abstinence over Weeks 9-24.

### Safety Analyses

**Unadjusted safety results:** The unadjusted safety results are presented in **Table 4**. The unadjusted analysis demonstrated that cytisinicline was associated with significantly lower odds of nausea compared with varenicline (OR, 0.17; 95% CI, 0.10-0.31 for inverse-variance, OR, 0.17; 95% CI, 0.10-0.30 for Mantel-Haenszel).

**Table 4.** Unadjusted Safety Results for Cytisinicline vs Varenicline

**Outcome**	**Cytisinicline vs Varenicline^a^, OR (95% CI)**	
**Inverse Variance**	**Mantel-Haenszel**	
All-cause treatment discontinuations	0.80 (0.53-1.21)	0.80 (0.53-1.21)
Any TEAE^b^	0.84 (0.61-1.15)	0.84 (0.61-1.15)
Discontinuations due to TEAEs	1.00 (0.41-2.44)	1.00 (0.41-2.45)
Insomnia	1.35 (0.78-2.35)	1.36 (0.78-2.35)
Abnormal dreams	0.81 (0.42-1.57)	0.82 (0.43-1.59)
Nausea	0.17 (0.10-0.31)*	0.17 (0.10-0.30)*

Abbreviations: CI, confidence interval; OR, odds ratio; TEAE, treatment-emergent adverse event.

*P < .001.

^a^Nonpsychiatric cohort.

^b^Total number of subjects with at least 1 adverse event.

**Adjusted safety results:** The adjusted safety results are summarized in **Table 5**. For all-cause treatment discontinuations, the number of subjects with TEAEs, and TEAEs of insomnia and abnormal dreams, no significant differences were observed for cytisinicline compared with varenicline. The aORs for these outcomes ranged from 0.78 to 0.89 for all-cause discontinuations, 0.78 to 0.88 for the number of subjects with TEAEs, and 0.84 to 1.43 for insomnia and abnormal dreams. A statistically significant difference was observed for nausea, where cytisinicline showed significantly lower odds of nausea compared with varenicline (Set 1: aOR, 0.18; 95% CI, 0.10-0.31; Set 2: aOR, 0.16; 95% CI, 0.09-0.29; Set 3: aOR, 0.13; 95% CI, 0.07-0.26).

**Table 5.** Adjusted Safety Results for Cytisinicline vs Varenicline

**Outcome**	**Cytisinicline vs Varenicline^a^, aOR (95% CI)**		
**Set 1 (Primary)**	**Set 2**	**Set 3**	
All-cause treatment discontinuations	0.78 (0.51-1.19)	0.89 (0.58-1.38)	0.84 (0.51-1.37)
Any TEAEs^b^	0.84 (0.61-1.16)	0.88 (0.63-1.22)	0.78 (0.54-1.15)
Discontinuations due to TEAEs	1.01 (0.41-2.47)	1.18 (0.46-3.00)	0.62 (0.23-1.71)
Insomnia	1.28 (0.72-2.26)	1.43 (0.79-2.58)	1.20 (0.62-2.31)
Abnormal dreams	0.87 (0.45-1.68)	0.91 (0.45-1.82)	0.84 (0.39-1.79)
Nausea	0.18 (0.10-0.31)*	0.16 (0.09-0.29)*	0.13 (0.07-0.26)*

Abbreviations: aOR, adjusted odds ratio; CI, confidence interval; HADS, Hospital Anxiety and Depression Scale; TEAE, treatment-emergent adverse event.

Note: Selected treatment effect modifiers Set 1 (race, cigarettes per day in the last 30 days), Set 2 (Set 1 + HADS anxiety score), and Set 3 (Set 2 + age + HADS depression score).

*P <.001.

^a^Nonpsychiatric cohort.

^b^Total number of subjects with at least 1 adverse event.

Discontinuations due to TEAEs (**Tables 4 and 5**) showed some variability across treatment effect modifiers, likely due to the low event rates in these outcomes, resulting in higher variance and uncertainty. Despite this, no outcome was found to change in directionality or significance due to adjusting for differences in investigated treatment effect modifiers.

## DISCUSSION

Cytisinicline 3 mg tid showed no statistically significant difference in efficacy compared with varenicline for smoking cessation after 12 weeks of treatment (Weeks 9-12), with significantly greater efficacy for maintaining longer-term smoking cessation at Weeks 9-24. This was demonstrated across all three sets of treatment effect modifiers investigated using the MAIC and unadjusted analyses. These analyses, over the extended time frame considered in the current analysis, suggest that cytisinicline may offer a longer-lasting effect in maintaining smoking cessation when compared with varenicline, and potentially indicates that cytisinicline is more effective in reducing the risk of later relapses.

The safety profiles of both treatments were similar across all three sets of treatment effect modifiers investigated, except for nausea. Nausea, a common adverse event associated with varenicline, was much less common among participants treated with cytisinicline. This finding was consistent across all three sets of treatment effect modifiers investigated. This is an important finding differentiating between cytisinicline and varenicline, given the impact that nausea can have on a patient’s adherence to treatment for a possible successful outcome. The tolerability of a smoking cessation medication plays a critical role in patient adherence and the overall effectiveness of the prescribed regimen.^45^ Adverse events associated with varenicline contribute to a 1.5-fold higher discontinuation rate than placebo; in the meta-analysis conducted by Drovandi et al, 9.13% of participants in the varenicline group, compared with 4.75% in the placebo group, had adverse effects, prompting dose reduction or temporary withdrawal.^24^ Catz et al reported similar findings from the COMPASS smoking cessation intervention trial, including which factors may influence smokers’ adherence to varenicline.^25^ This study demonstrated that one of the most frequent reasons for stopping varenicline early was having adverse events with this treatment.^25^ Over half of smokers (52%) in the study who were no longer taking varenicline at the 21-day follow-up indicated that the reason for stopping was due to adverse events.^25^

Challenges of using MAIC include the risk of overmatching when adjusting for baseline characteristics. This risk was mitigated by prespecifying potential effect modifiers identified as significant or potentially impactful in prior studies. Also reported was the estimated effective sample size for the adjusted analyses to enable assessment of the risk of inflated uncertainty. A limitation of this analysis is that this comparison was not part of a head-to-head RCT and relied on an indirect comparison between the two treatments. Conducting head-to-head RCTs presents challenges, including the inability to blind treatments, trial costs, and comparator choice, especially in an evolving treatment landscape. As a result, the use of existing, comparative data from ITCs has been shown to represent a valuable resource, resulting in clinical insights that reflect current practice.^46–48^ A notable limitation of standard ITC methods is the assumption of homogeneity in treatment effect modifiers. In the primary analysis, this was addressed by adjusting for relevant treatment effect modifiers, enabling a comparison of cytisinicline’s estimated treatment effect against the reported effect of varenicline in the EAGLES nonpsychiatric cohort. The adjusted and unadjusted efficacy analyses showed consistent results across both outcomes. Furthermore, the amount of uncertainty around the results was similar in both analyses, indicating that the populations under investigation (the pooled ORCA data and the EAGLES nonpsychiatric cohort) were sufficiently comparable, and that treatment effect modifiers had minimal influence on the results.

## CONCLUSIONS

In this indirect comparison of efficacy and safety for cytisinicline compared with varenicline, cytisinicline exhibited a lower risk of later relapse during the post-treatment phase. Additionally, the safety profile of cytisinicline included a lower incidence of nausea vs varenicline, suggesting that cytisinicline may represent a more tolerable option for smoking cessation. This improved tolerability could enhance patient adherence, supporting the overall success of smoking cessation efforts. Future research or head-to-head RCTs should seek to further validate these findings and strengthen the case for cytisinicline as a long-term solution for smoking cessation, especially for individuals at an increased risk of relapse or those who had nausea from alternative medications.

### Conflict of Interest Disclosures

M.R. and R.P. are employees of Achieve Life Sciences. D.R. is an employee of Medical Decision Modeling. C.J. was an employee of Achieve Life Sciences at the time this analysis was performed.

### Meeting Presentation

A poster based on this study was presented at the American Thoracic Society 2026 International Conference in Orlando, Florida.

## Supplementary Material

Graphical Abstract

Plain Language Summary
