# Supplementary material for: Comparative Effectiveness of Cytisinicline and Varenicline for Smoking Cessation: A Matching-Adjusted Indirect Comparison (MAIC)
Source: J Health Econ Outcomes Res. 2026 May 20;13(1):182–8. doi: 10.36469/001c.160017 (PMC13196912; doi:10.36469/001c.160017)

Comparative effectiveness of cytisinicline and varenicline for smoking cessation: A matching-adjusted indirect comparison (MAIC)

AIM

To compare 2 treatments that can help people quit smoking

TREATMENTS COMPARED

Cytisinicline
(3 mg three times daily for 12 weeks, from ORCA-2 and ORCA-3 studies)

VS.

Varenicline
(1 mg twice daily for 12 weeks [Week 1 uptitration, Weeks 2–12 at full dose], from EAGLES study)

POPULATION

- Adults
- Smokers: ≥10 cigarettes daily
- Motivated to quit

METHODS

- MAIC analysis of clinical trial data
- Compared the effectiveness and side effects of the 2 treatments

Cytisinicline versus varenicline: **Which worked better?**

People who quit smoking and stayed smoke-free over time
Odds ratio 95% CI (significant if do not cross 1)

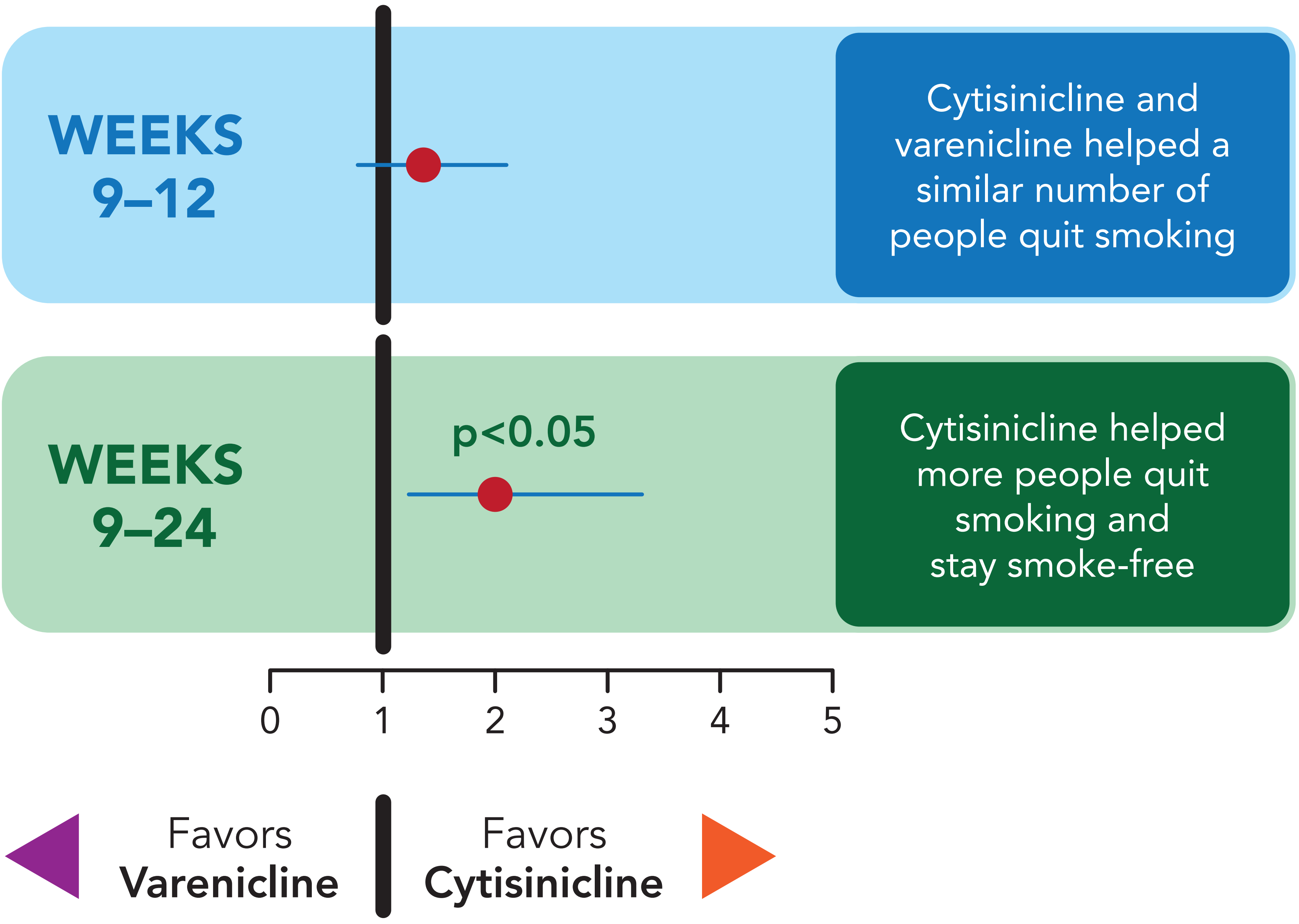

Cytisinicline versus varenicline: **Which had fewer side effects?**

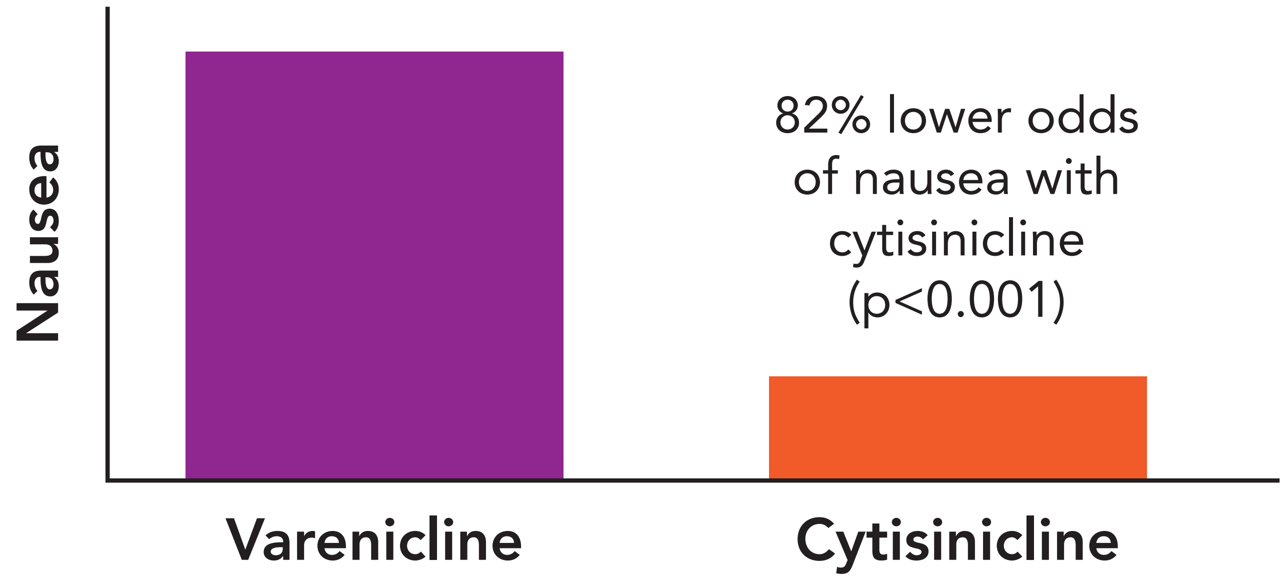

Supplement: Graphical Abstract [file jheor_2026_13_1_160017_341381.pdf]
