# Supplementary material for: Comparative Effectiveness of Cytisinicline and Varenicline for Smoking Cessation: A Matching-Adjusted Indirect Comparison (MAIC)
Source: J Health Econ Outcomes Res. 2026 May 20;13(1):182–8. doi: 10.36469/001c.160017 (PMC13196912; doi:10.36469/001c.160017)
Supplement: Plain Language Summary [file jheor_2026_13_1_160017_345514.pdf]

PLAIN LANGUAGE SUMMARY

Comparing Two Quit-Smoking Medicines: Cytisinicline and Varenicline

What is this study about?

This study compared two medicines that can help people quit smoking. The two medicines are called cytisinicline and varenicline. Cytisinicline works by reducing cravings and withdrawal symptoms that can make quitting smoking difficult. Cytisinicline is currently under FDA review in the United States. Varenicline is already an approved quit-smoking medicine available in the United States. Researchers wanted to know:

- Which medicine works better?
- Which medicine has fewer side effects?

What were the results?

At 12 weeks, both medicines had helped a similar number of people quit smoking. However, looking at how many people were able to refrain from smoking between 9 and 24 weeks after starting treatment, more people who took cytisinicline had stayed smoke-free compared with those on varenicline. People who took cytisinicline had 82% lower odds of feeling sick (nausea) than those taking varenicline.

What were the limitations?

Because the two medicines were tested in separate studies, the researchers used a statistical method to match the study participants as closely as possible for comparison of results.

What do the results mean?

Both cytisinicline and varenicline are effective medicines that can help people quit smoking. The results suggest that cytisinicline helped more people to stay smoke-free after taking the treatment. Cytisinicline also caused significantly less nausea compared with varenicline. These findings suggest cytisinicline may be a promising new option for people trying to quit smoking.
